# Arbuscular Mycorrhizal Fungi Regulate Lipid and Amino Acid Metabolic Pathways to Promote the Growth of *Poncirus trifoliata* (L.) Raf

**DOI:** 10.3390/jof10060427

**Published:** 2024-06-18

**Authors:** Yihao Kang, Gratien Twagirayezu, Jie Xu, Yunying Wen, Pengxiang Shang, Juan Song, Qian Wang, Xianliang Li, Shengqiu Liu, Tingsu Chen, Tong Cheng, Jinlian Zhang

**Affiliations:** 1 Microbiology Research Institute, Guangxi Academy of Agricultural Sciences, Nanning 530007, China; kangyh0810@163.com (Y.K.); jiexu0331@163.com (J.X.); 14777282841@163.com (Y.W.); w240940688@126.com (J.S.); wangqian589@126.com (Q.W.); chents001@gxaas.net (T.C.); 2 School of Public Health, Xiamen University, Xiamen 361102, China; px_shang@126.com; 3 State Key Laboratory of Environmental Geochemistry, Institute of Geochemistry, Chinese Academy of Sciences, Guiyang 550002, China; tgratien0@gmail.com; 4 University of Chinese Academy of Sciences, Beijing 100049, China; 5 Guangxi Academy of Specialty Crops, Guilin 541004, China; xlfd003@126.com (X.L.); lsqfine1024@163.com (S.L.); 6 School of Life Sciences, Xiamen University, Xiamen 361102, China; tcheng@xmu.edu.cn

**Keywords:** arbuscular mycorrhizal fungi, symbiosis, citrus, genetic pathways, metabolomics

## Abstract

Arbuscular mycorrhizal (AM) fungi can enhance the uptake of soil nutrients and water by citrus, promoting its growth. However, the specific mechanisms underlying the action of AM fungi in promoting the growth of citrus were not fully elucidated. This study aimed to explore the role of AM fungi *Funneliformis mosseae* in the regulatory mechanisms of *P. trifoliata* growth. Pot experiments combined with non-targeted metabolomics methods were used to observe the growth process and changes in metabolic products of *P. trifoliata* under the conditions of *F. mosseae* inoculation. The results showed that *F. mosseae* could form an excellent symbiotic relationship with *P. trifoliata*, thereby enhancing the utilization of soil nutrients and significantly promoting its growth. Compared with the control, the plant height, stem diameter, number of leaves, and aboveground and underground dry weight in the *F. mosseae* inoculation significantly increased by 2.57, 1.29, 1.57, 4.25, and 2.78 times, respectively. Moreover, the root system results confirmed that *F. mosseae* could substantially promote the growth of *P. trifoliata*. Meanwhile, the metabolomics data indicated that 361 differential metabolites and 56 metabolic pathways were identified in the roots of *P. trifoliata* and were inoculated with *F. mosseae*. This study revealed that the inoculated *F. mosseae* could participate in ABC transporters by upregulating their participation, glycerophospholipid metabolism, aminoacyl tRNA biosynthesis, tryptophan metabolism and metabolites from five metabolic pathways of benzoxazinoid biosynthesis [mainly enriched in lipid (39.50%) and amino acid-related metabolic pathways] to promote the growth of *P. trifoliata.*

## 1. Introduction

Citrus is one of the most economically significant crops globally, planted in over 140 countries and regions [1]. According to data published by the World Citrus Organization, during the summer and winter seasons of 2021 and 2022, 44.6 million tons of citrus was produced in China, accounting for 28% of the world’s total production and ranking first in the world (Citrus World Statistics-WCO-November 2022). Citrus ranks as the third largest traded agricultural product in the world after corn and wheat [2]. Recently, due to long-term continuous cropping and use of inorganic fertilizers, the soil has been continuously acidified and hardened, thereby affecting the nutrient supply from the soil to the aboveground part, ultimately reducing the growth and even yield of citrus [3]. In actual production, most citrus rootstocks, such as *Citrus aurantium* L., *P. trifoliata* and *C*. *reticulata* Blanco, have very little or no development of root hair structures during field cultivation, impeding the uptake capacity for water and nutrients from the soil environment. The inappropriate agricultural management system further exacerbates the nutritional supply problem of citrus fruits. Improving the utilization efficiency of nutrients in the soil by citrus and ensuring the high-quality and high-yield growth of citrus has become a bottleneck problem for the sustainable development of healthy agriculture, which is a thorny problem to be solved in achieving rural revitalization.

Research has shown that the inoculation of AM fungi can form a symbiotic relationship with citrus, improving the water and nutrient supply of the host plant citrus through hyphal development [4]. AM fungi are specialized symbiotic fungi that form symbiosis with most land plants [5,6]. They demonstrate a favorable impact on the soil structure [7], nutrient uptake and host plants growth [8,9] and alleviate biotic and abiotic stresses on host plants [10,11].

Recently, numerous reports were on promoting citrus growth by inoculation with AM fungi. Niu et al. [12] have reported that the inoculation of *G. mossase* promoted the development of orange, which comprehensively improved plant height, chlorophyll, photosynthesis, and aboveground and underground dry matter. Zhang et al. [13] detected positive results in physiological indicators such as plant height, stem thickness, number of leaves, and petiole length in the seedlings of *P. trifoliata* with the inoculation of *Redeckera fulvum* and *F. mosseae*. Meanwhile, field cultivation experiments further showed that the inoculation of AM fungal significantly enhanced the mycorrhizal infection rate, hyphal density, root vitality, and content of ascorbic acid-related proteins in citrus and improved fruit indicators such as soluble solid content, longitudinal and transverse diameter, and single fruit weight. Moreover, the effect of mixed inoculation was better than that of single inoculation treatment [14]. After the inoculation with AM fungi, the uptake area of water and soil nutrients by the host plant is expanded through hyphae to promote the growth of the host plant [15]. Simultaneously, the inoculation of AM fungus improves crop photosynthetic parameters and enhances plant leaf photosynthetic rate and photosynthetic capacity [16]. It also alters root fatty acid composition and saturation, enhancing the plant’s antioxidant defense system and adaptability to stressful environments, thereby promoting overall plant growth [17,18].

Although the above studies have comprehensively investigated the epigenetic and physiological responses of citrus and have also explained the promotion of citrus growth by AM fungal inoculation from a macro perspective, there is a lack of corresponding reports on its molecular mechanism [19]. With the continuous advancement of molecular technology, metabolomics has gradually emerged as a widely used and highly recognized analytical method for studying plant growth and development [20,21]. Therefore, this study aimed to use metabolomics analysis to investigate the changes in metabolites of AM fungi in the root system of citrus seedlings.

## 2. Materials and Methods

### 2.1. Experimental Materials

#### 2.1.1. The Used Citrus Plant

The tested citrus rootstock is *P. trifoliata* and its seeds were taken from the Guangxi Special Crop Research Institute, Guilin, Guangxi, China.

#### 2.1.2. The Used AM Fungi Isolate

The AM fungus *F. mosseae* (formerly *Glomus mosseae*) hypothesized in this study was isolated from a citrus orchard by the Institute of Microbiology of the Guangxi Academy of Agricultural Sciences, Fuchuan, Guangxi, China. After single spore cultivation, it was stored at the Subtropical Arbuscular Mycorrhizal Fungal Resource Preservation Center (amf-cmcc.com) with the isolate number BGSC-71. A mixture of plant roots, AM fungal spores (Figure S1), and growth medium was stored in a refrigerator at 4 °C. The fungal inoculum was propagated using corn as the host plant. The procedure involved filling a 1000 mL plastic pot with a mixture of sterilized sand and zeolite (1:1, *v*/*v*), placing 50 g of AM fungal inoculum on the surface of the growth medium, disinfecting the inoculum with 10% H_2_O_2_ for 10 min, rinsing it five times with sterile water, and then placing sterilized corn seeds on the inoculum, covering them with a 2 cm layer of sterilized substrate. The pots were placed in a greenhouse under controlled conditions, with a temperature of approximately 25–30 °C, humidity of 60–70%, natural light, watering with distilled water every 1–2 days, and bi-weekly fertilized with Hoagland’s low-phosphorus solution. After four months, the pots were left to dry for two weeks, after which the roots and growth medium were harvested. The cultures were considered successfully established when the growth substrate contained spores at various developmental stages, and the stained roots were colonized by mycorrhizal structures. This mixture of plant roots, AM fungal spores, and growth medium constitutes the AM fungal inoculum, with a spore count of more than 50 mature spores per gram of inoculum.

#### 2.1.3. The Used Soil and Substrates

The used soil was taken from the uppermost layer (0–20 cm) of The Garden of Fuchuan, Guangxi, by the Institute of Microbiology of Guangxi Academy of Agricultural Sciences. It was then carefully processed: stones and plant root residues were removed, and the soil was naturally dried before being thoroughly mixed. Afterward, the mixture underwent screening through a 5 mm nylon mesh screen and was sterilized using 10 kGy of Cobalt-60 radiation. The basic physicochemical properties of the used soil are presented in Table 1. The used substrate was composed of a mixture of seedling organic matter, sand, and soil mixed in a ratio of 2:1:1. After that, it was placed in a sterilization bag and subjected to high-pressure steam sterilization at a temperature of 121 °C for 120 min.

### 2.2. Experimental Design

The cultivation experiment used *P. trifoliata* as the cultivation material and was carried out in the core experimental area of the Guangxi Academy of Agricultural Sciences. In the experiment, two treatments were implemented: inoculation with AM fungal treatment (AMF) and a non-inoculation treatment (CK). The number of replicates for each treatment was 12, of which 6 were used for root infection and metabolome studies, and the remaining 6 were assigned for biomass measurements. Plump seeds of *P. trifoliata* were selected and soaked in a 1 ‰ potassium permanganate solution for 2 min before germination under 25–30 °C conditions with a maintained humidity of 95%. After germination, the seeds were sown in a seedling pot for cultivation under conditions of 25–30 °C and a humidity range of 60–70%. When the *P. trifoliata* seedlings reached a height of 10 cm, plants showing consistent growth were selected and transplanted into pots with a top diameter of 15 cm, pot height of 12.5 cm, and bottom diameter of 11 cm. A piece of paper was placed at the bottom of the basin to prevent substrate or water leakage before the substrate was prepared. After that, half of the basin was filled with the substrate. According to the experimental design, 300 spores per pot of AM fungal inoculum were added to the middle of the pot substrate, of which 50 spores were used as the number of spores per gram of the test strain. The *P. trifoliata* seedlings were placed on the AM fungal inoculum so that their roots were entirely in contact with it. Again, the substrate was added to 3/4 of the pot, ensuring complete coverage of the roots. The control group was provided with an equivalent quantity of sterilized substrate. In the initial stage, the roots were watered to ensure thorough substrate saturation. Subsequently, every two weeks, regular watering was maintained with Hoagland nutrient solution (200 mL/pot). The incubation experiment lasted for 7 months until the plant was collected.

### 2.3. Experimental Methods

#### 2.3.1. Determination of Plant Growth Indicators

At the beginning of the cultivation experiment for 3, 5, and 7 months, the distance from the soil surface to the top of the plant was measured using a rule as the plant height. The stem diameter at a distance of 1 cm from the soil surface was determined as the stem diameter. The cotyledons of each citrus plant were counted, excluding all other mature leaves, and they were recorded as the number of leaves.

#### 2.3.2. Root Infection Determination

At plant harvesting, roots were collected, rinsed with clean water, and then soaked in 50% alcohol for storage and testing. As outlined by Li et al. [22], the root staining method was utilized with enhancements. The root system was fixed in 50% alcohol, thoroughly rinsed with water, and placed in a tissue embedding box. Subsequently, 20% KOH was added, followed by a water bath at 90 °C for 0.5 h. The treated roots were washed three times with water to maintain moisture content. Decolorization was achieved by adding alkaline H_2_O_2_ for 2 h, followed by rinsing with clean water three times. Acidification was performed using 5% glacial acetic acid for 2 h before introducing a 5% ink vinegar dye solution. The roots were stained in a 66 °C water bath for 1 h, rinsed 3–5 times with clean water, and soaked in clean water for at least 12 h to ensure decolorization for subsequent use.

#### 2.3.3. Sampling for Metabolomics Analysis

In the seventh month after inoculation, the aboveground part of the plant was cut, and the root system was unearthed and thoroughly washed. After drying with dust-free paper, they were cut into small pieces with scissors and put into a cryopreservation tube. After that, they were promptly placed in a liquid nitrogen box and expeditiously sent to the laboratory. Upon arrival, they were stored in a refrigerator at −80 °C for subsequent testing.

The non-targeted metabolomics methods were used to analyze the primary and secondary metabolites, which were identified using an ultra-high performance liquid chromatography–tandem Fourier transform mass spectrometry (UHPL-Q Exactive) system from Shanghai Meiji Biopharmaceutical Technology Co., Ltd. The variable weight values (VIP) and *p*-values from the Student’s *t*-test obtained through the OPLS-DA model were utilized to select differential metabolites. Metabolites with VIP > 1 and *p* < 0.05 were marked as significantly different metabolites. Differential metabolites were annotated through the KEGG database (https://www.kegg.jp/kegg/pathway.html, accessed on 24 April 2024) for metabolic pathway analysis to obtain the metabolic pathways involved in differential metabolites.

#### 2.3.4. Root Scanning Analysis

**Figure 1:**
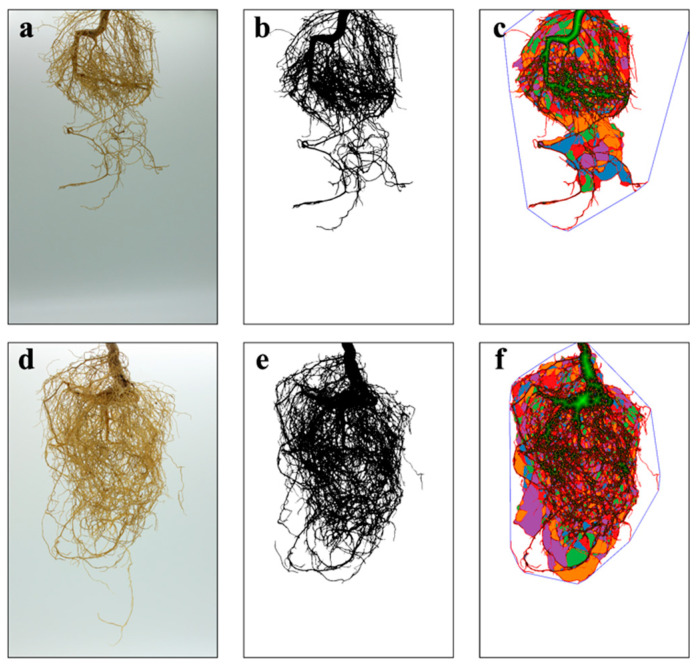
The growth and scanning of the root system of *P. trifoliata* inoculated without (**a**–**c**) and with AM fungi (**d**–**f**).

Photos were taken with a camera after cleaning and drying the root system with a dust-free cloth. The root characteristics of each treatment, including total root length, root surface area, root volume, and root diameter (Figure 1), were analyzed using RhizoVision Analyzer V1.

#### 2.3.5. Biomass Measurement

After completing the root scanning analysis photo collection, the roots were individually placed in paper bags, dried in an oven at 105 °C for 30 min, and dried at 70 °C until a constant weight was achieved. The aboveground and underground biomasses were calculated separately.

#### 2.3.6. Data Analysis

All data were checked for normality using the Shapiro–Wilk test, and Levene’s test was used to test the equality of variance. The mean and standard error of experimental data were calculated using Microsoft Excel 2010. Single-factor analysis of variance was conducted using SPSS (Version 25.0). Duncan’s method was used for multiple comparisons. Any statistical tests resulting in a *p* < 0.05 were considered statistically significant. All the metabolome visualizations were assessed using an online platform (www.majorbio.com). The graphs were drawn using Origin 2021.

## 3. Results

### 3.1. Physiological Indicators of P. trifoliata

**Figure 2:**
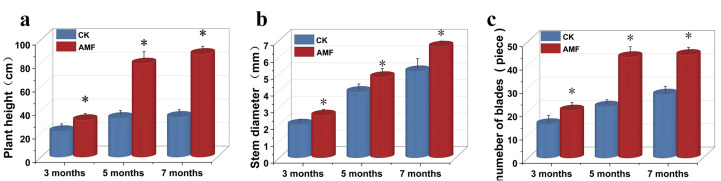
Plant height (**a**), stem diameter (**b**), and number of leaves (**c**) of orange *P. trifoliata* in the CK and AMF treatments at different times after inoculation. All data are presented as the mean ± standard deviation (n = 6). * represents a significant difference between treatments (*p* < 0.05).

**Figure 3:**
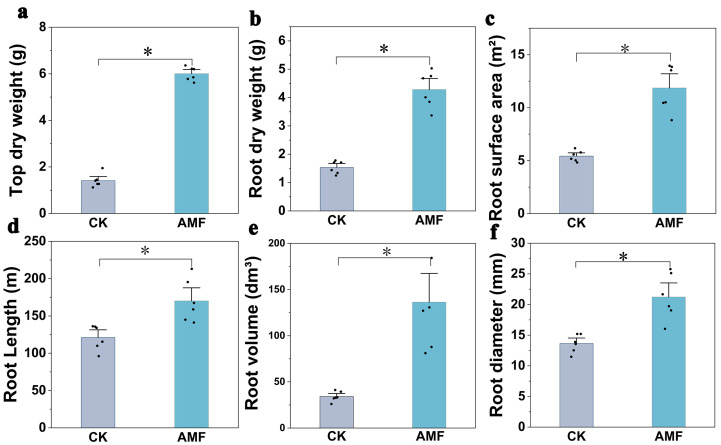
Top dry weight (**a**), root dry weight (**b**), root diameter (**c**), root length (**d**), root volume, (**e**) and root surface area (**f**) in CK and AMF treatment. The labels of CK and AMF represent without (control) and with AM fungi (*F. mosseae*). All data are presented as the mean ± standard deviation (n = 6). * represents a significant difference between treatments (*p* < 0.05).

The inoculation with *F. mosseae* could significantly improve the growth (Figure S2) and root system development (Figure S3) of *P. trifoliata*. At the end of 3 months, the plant height in the AMF inoculation treatment was significantly 1.43 times higher than that in the CK, and the stem diameter in the AMF treatment (2.51 ± 0.20 cm) was higher (*p* < 0.05) than that in the CK (1.97 ± 0.16 cm). Meanwhile, the leaf number increased from 14.5 ± 2.51 cm in the control to 20.33 ± 2.25 cm in the AMF treatment. Similarly, there was a significant improvement in the AMF treatment at 5 and 7 months after incubation compared with the CK (Figure 2). For the dry weight of aboveground parts, dry weight of underground parts and various root traits, the AMF treatment exhibits significantly higher values than those in the CK (Figure 3).

### 3.2. Metabolomic Characteristics of P. trifoliata

#### 3.2.1. Metabolite Detection and Classification

**Figure 4:**
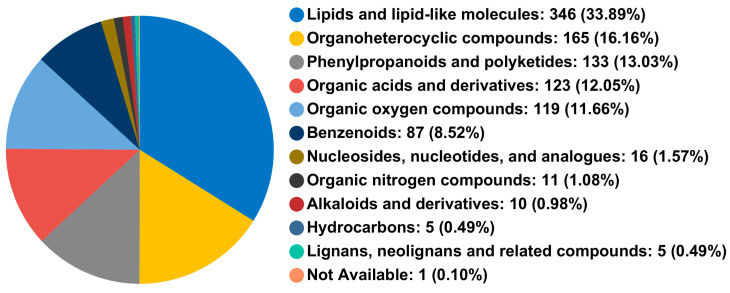
A pie chart of the classification of metabolites in the root samples of *P. trifoliata* is at the superclass level. Different colors in the pie chart represent different HMDB classifications; the area means the relative proportion of metabolites.

As displayed in Figure 4, metabolites with positive and negative ion modes were extracted and identified using UHPLC-TOF-MS/MS technology. After data preprocessing, 1021 metabolites were detected, including 602 in positive and 419 in negative ion modes. At the superclass level, these metabolites were classified into 11 categories.

#### 3.2.2. Analysis of Differential Metabolites

**Figure 5:**
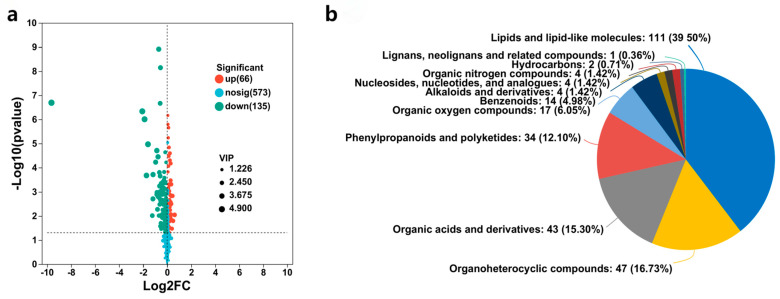
(**a**) Volcano map for the positive and negative ion merging mode. Up represents significantly upregulated metabolites, down represents significantly downregulated metabolites, and nosing represents metabolites with no differences; (**b**) pie chart of differential metabolite classification at the superclass level, where different colors represent different HMDB classifications, and the area means the relative proportion of metabolites in the classification.

Based on VIP > 1 and *p* < 0.05, 361 differential metabolites were screened under the combination of positive and negative ions mode. Among them, 128 metabolites are significantly upregulated, whereas 233 are downregulated considerably, as presented in Figure 5a. The classifications of differential metabolites at the superclass level are well-illustrated in Figure 5b.

#### 3.2.3. KEGG Enrichment Analysis

**Figure 6:**
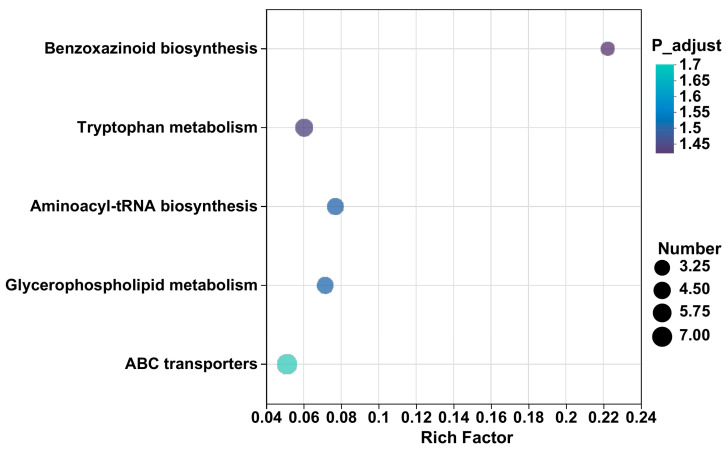
Pathways for differential metabolites between CK and AMF treatment.

As indicated in Figure 6, 361 differential metabolites are identified, and 56 metabolic pathways related to 51 plant metabolisms are annotated. Among them, five metabolic pathways, including ABC transporters, glycerophospholipid metabolism, aminoacyl tRNA biosynthesis, tryptophan metabolism, and benzoxazinoid biosynthesis, are significantly enriched (*p* < 0.05). In the ABC transport pathway, seven metabolites, including L-threonine, Myo-inositol, L-histidine, L-serine, sucrose, uridine, and betaine, are observed to be upregulated in the AMF treatment compared with the control based on a corrected *p*-value (*p* < 0.05) (Table 2). Compared with the control, the inoculation of AM fungi significantly upregulates three metabolites, including dimethylethanolamine, phosphocholine, and L-serine in glycerol phospholipid metabolism pathways. Four metabolites are upregulated in the aminoacyl tRNA biosynthesis pathway, including L-histidine, L-serine, L-tyrosine, and L-threonine. In the tryptophan metabolism pathway, five metabolites, including indoxyl, formyl-5 hydroxykynurenamine, 3-methyliodoxindole, 5-hydroxyindoleacetic acid, and N-methyltryptamine are significantly upregulated. Moreover, two metabolites—3-hydroxyindolin-2-one and oxindole—show increased levels in the metabolic pathway of benzoxazinoid biosynthesis. However, PE 18:3 (6Z, 9Z, 12Z)/20:5 (5Z, 8Z, 11Z, 14Z, 17Z) is significantly downregulated.

## 4. Discussion

### 4.1. Effects of AM Fungi Inoculation on Physiological Indicators of P. trifoliata

Recently, several studies have focused on the growth effects of citrus rootstocks inoculated with AM fungi [12,13]. A study conducted by Edriss et al. [23] has shown an increase in the total dry weight of four citrus seedlings after the inoculation of AM fungi in comparison with non-inoculated seedlings. Another study carried out by Wang et al. [24] has revealed a significant growth of seedlings inoculated with AM fungi relative to the control group. It has shown that the differences in the number of fibrous roots and fresh weight of the underground parts of the measured items were significantly higher than those in the control group. In this study, inoculation with *F. mosseae* significantly increased the plant height, stem diameter, leaf number, and biomass of *P. trifoliata* (Figure 2, Figure 3 and Figure 4). At 7 months after inoculation, the plant height, stem diameter, number of leaves, aboveground dry weight, and underground dry weight in the treatment with AM fungi (AMF) were 2.57, 1.29, 1.57, 4.25, and 2.78 times higher, respectively, compared with the CK. The above results also confirmed that AM fungi inoculation could promote citrus growth. Although several studies have shown that AM fungi inoculation has a significant stimulating effect on the growth of citrus [13], there are also reports showing that inoculation with *G. clarum* did not show any impact on the plant height, stem thickness, aboveground dry weight, and underground weight of *P. trifoliata* [25]. Even the inhibitory effects of *G. versiforme* inoculation on plant height, stem diameter, and biomass of red-orange tissue cultured seedlings were reported [26]. The possible reason is that under limited nutrient conditions, the development of AM fungi not only requires the host plant to provide carbohydrates but also competes with the host plant for nutrients, resulting in no effect or even negative effect on the growth of the host plant [27]. On the other hand, the regulation of host plant growth through AM fungal inoculation is influenced by the specific genus and species of the inoculated fungi [28].

In addition, the infection of AM fungi also induces changes in the root morphology of host plants. Yao et al. [29] found that inoculation with AM fungi significantly promoted the formation of higher-order secondary roots in the root system of *P. trifoliata*, inducing more young and new roots, but significantly reduced the total length, volume, and surface area of the roots. However, no significant effect was observed on the primary root length and average root diameter of *P. trifoliata*. This study showed that inoculation with *F. mosseae* could significantly improve the root characteristics of *P. trifoliata*. At 7 months after inoculation, the root diameter, total root length, root volume, and root surface area in AM fungi inoculated treatment were 1.56, 1.40, 3.99, and 2.19 times higher than those in the CK. This is corroborated by the study results conducted by Li et al. [30] Improving root system configuration can help host plants absorb more water and nutrients from the soil.

### 4.2. Regulation of Inoculation with AM Fungi on Metabolic Pathways in P. trifoliata

AM fungi not only improve plant nutrition and health but also induce physiological changes in plants, affecting the metabolic processes of host plants. Metabolic network regulation plays a vital role in plants [31]. In this study, five pathways, including ABC transporters, glycerophorid metabolism, aminoacyl tRNA biosynthesis, tryptophan metabolism, and benzoxazinoid biosynthesis, were significantly enriched in the AMF treatment compared with the CK (Table 2).

A study conducted by Wu et al. [32] has revealed that the ABC transporter protein family is involved in the tissue transport of various plant growth and development stages, including plant hormones and secondary metabolites during plant growth and development. Seven metabolites (L-threonine, Myo-inositol, L-histidine, L-serine, sucrose, uridine, and betaine) were significantly upregulated in this study compared with CK. This shows that AM fungi inoculation has many positive effects on regulating cell tissue differentiation and promoting germination [33], improving the ability of plants to resist stress [34] and the ability to provide energy and build a carbon framework for plant material metabolism [35].

Fatty acids are the primary carbon source transmitted by plants to AM fungi [36]. Due to the lack of genes encoding the fatty acid synthase I subunit in AM fungi, a symbiotic system between AM fungi and plant roots could induce plant lipid synthesis and transport lipids to AM fungi to maintain colonization [37]. In this study, the percentage of mycorrhizal colonization and arbuscular abundance significantly increased in the AMF treatment compared with CK treatments (Figure S4). In the early stage of AM fungal infection, many genes related to transport, transcriptional regulation, and tissue lipid metabolism are differentially expressed [38]. These genes only exist in the host plants of AM fungi [39]. This study showed that among the differential metabolites in the root system of *P. trifoliata* inoculated with AM fungi, lipids and lipid molecules accounted for the most significant proportion (39.50%), and AM fungal inoculation increased the proportion of lipids and lipid molecules in the metabolites.

The aminoacyl tRNA biosynthetic metabolic pathway is related to biological translation activities and involves the biosynthesis of various amino acids. Amino acids are proteins and enzyme components and serve as signaling molecules to regulate plant environmental stress [40]. Compared with non-mycorrhizal plants, arbuscular mycorrhizal plants absorb more amino acids from soil [41]. Previous studies have reported that the amino acid content in plants increases, decreases, or remains unchanged due to inoculation with AM fungi [42,43]. In this study, the expression levels of four amino acids (L-histidine, L-serine, L-tyrosine, L-threonine) in the biosynthetic metabolic pathway of aminoacyl tRNA were significantly upregulated, indicating that AM fungal inoculation is beneficial for the transport of amino acids in the roots of *P. trifoliata*. Based on the above analysis, the inoculation of AM fungi could promote the absorption of soil nutrients (Table 3) in citrus by regulating metabolic channels, thereby promoting *P. trifoliata* growth.

### 4.3. Correlation between the Differential Metabolites of P. trifoliata Roots and the Growth Indexes

**Figure 7:**
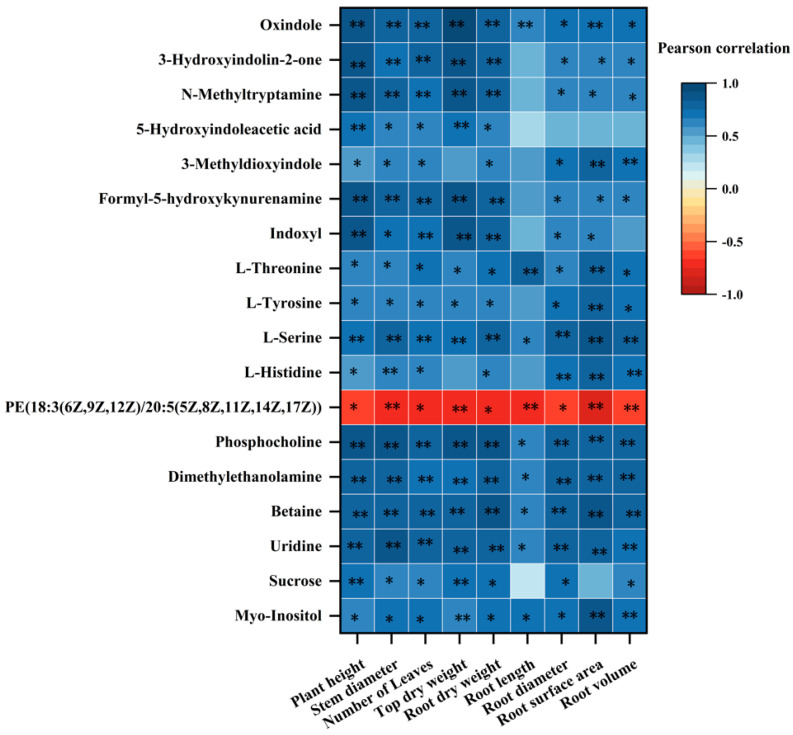
Heat map of correlation between the differential metabolites of roots and the growth indexes of *P. trifoliata* in CK and AMF treatment. ‘**’ represents a significant correlation of 0.01 level, whereas ‘*’ represents a significant correlation of 0.05 level.

As shown in Figure 7, except for PE (18:3 (6Z, 9Z, 12Z)/20:5 (5Z, 8Z, 11Z, 14Z, 17Z)) and the growth indicators of *P. trifoliata*, the other different metabolites were positively correlated with the growth of *P. trifoliata*. Among these metabolites, L-serine, phosphocholine, dimethylethanolamine, betaine, and uridine had a highly significant positive correlation with various growth indices of *P. trifoliata*, such as a significant positive correlation with root length and others. These five metabolites are all enriched in ABC transporters or glycerophospholipid metabolism. Therefore, inoculated AM fungi primarily regulate these two metabolic pathways to promote the growth of *P. trifoliata*.

## 5. Conclusions

The results revealed that inoculation of *F. mosseae* significantly enhanced various physiological indicators in *P. trifoliata*, indicating the formation of a symbiotic relationship. This was evidenced by improvements in nutrient utilization and growth promotion. Specifically, the inoculation significantly increased plant height, stem diameter, leaf number, and aboveground and underground dry weights. Metabolomic analysis identified 1021 detected metabolites, with 361 identified as differential metabolites between the CK and AMF treatments. These metabolites were associated with 56 metabolic pathways, revealing insights into the regulatory mechanisms induced by AM fungi. Notably, pathways such as ABC transporters, glycerophospholipid metabolism, aminoacyl tRNA biosynthesis, tryptophan metabolism, and benzoxazinoid biosynthesis were significantly enriched, highlighting the involvement of AM fungi in crucial metabolic processes, including nutrient transport, lipid metabolism, amino acid biosynthesis, and stress response. Overall, this study sheds light on the molecular mechanisms underlying the positive effects of AM fungi on citrus growth and development, particularly *F. mosseae*. The findings contribute to understanding how AM fungi participate in metabolic pathways to enhance nutrient uptake and promote overall growth in *P. trifoliata*. This research has implications for agricultural practices aimed at improving citrus cultivation and addressing challenges related to soil nutrient depletion and plant growth.

## Figures and Tables

**Table 1:** Physicochemical properties of the used soil. All data are presented as the mean ± standard deviation (n = 6).

Parameters	Soil
pH	6.73 ± 0.02
Organic matter (g/kg)	79.99 ± 2.58
Total phosphorus (g/kg)	0.60 ± 0.01
Alkali-hydrolyzed nitrogen (mg/kg)	95.16 ± 3.12
Available phosphorus (mg/kg)	67.75 ± 2.74
Available potassium (g/kg)	708.50 ± 20.11

**Table 2:** Significant enrichment pathways of differential metabolites between CK and AMF treatment. Note: The metabolic pathways in the table were obtained through KEGG pathway enrichment analysis. The differential metabolites in the table refer to those involved in the corresponding metabolic pathways. The enrichment factor is the ratio of the number of differential metabolites in the table to the number of all metabolites involved in the related metabolic pathway. The *p*-value is the corrected *p*-value.

Pathway Description	Rich Factor	*p*-Value	Differential Metabolites	Up/Down
ABC transporters	0.05	0.02	L-threonine	up
			Myo-inositol	up
			L-histidine	up
			L-serine	up
			Sucrose	up
			Uridine	up
			Betaine	up
Glycerophospholipid metabolism	0.07	0.03	Dimethylethanolamine	up
			Phosphocholine	up
			L-serine	up
			PE(18:3(6Z,9Z,12Z)/20:5(5Z,8Z,11Z,14Z,17Z))	down
Aminoacyl-tRNA biosynthesis	0.08	0.03	L-histidine	up
			L-serine	up
			L-tyrosine	up
			L-threonine	up
Tryptophan metabolism	0.06	0.04	Indoxyl	up
			Formyl-5-hydroxykynurenamine	up
			3-methyldioxyindole	up
			5-hydroxyindoleacetic acid	up
			N-methyltryptamine	up
Benzoxazinoid biosynthesis	0.22	0.04	3-hydroxyindolin-2-one	up
			Oxindole	up

**Table 3:** Physicochemical properties of CK and AMF treatment substrate. All data are presented as the mean ± standard deviation (n = 6).

Parameters	CK Treatment Substrate	AMF Treatment Substrate
pH	6.66 ± 0.02	6.70 ± 0.01
Organic matter (g/kg)	97.50 ± 5.34	80.51 ± 1.79
Total phosphorus (g/kg)	0.77 ± 0.01	0.69 ± 0.01
Alkali-hydrolyzed nitrogen (mg/kg)	103.16 ± 1.62	94.03 ± 1.2
Available phosphorus (mg/kg)	77.09 ± 3.66	91.22 ± 1.95
Available potassium (g/kg)	714.67 ± 20.56	660.00 ± 11.12

## Data Availability

Data are contained within the article and supplementary materials.

## Supplementary Materials

The following supporting information can be downloaded at: https://www.mdpi.com/article/10.3390/jof10060427/s1.
