# Supplementary material for: Arbuscular Mycorrhizal Fungi Regulate Lipid and Amino Acid Metabolic Pathways to Promote the Growth of Poncirus trifoliata (L.) Raf
Source: J Fungi (Basel). 2024 Jun 18;10(6):427. doi: 10.3390/jof10060427 (PMC11204456; doi:10.3390/jof10060427)
Supplement: Supplementary file 1 [file jof-10-00427-s001.zip › jof-3006598-supplementary.pdf]

Supplemental materials for

Arbuscular Mycorrhizal Fungi Regulate Lipid and Amino Acid Metabolism Pathways to Promote the Growth of *Poncirus trifoliata* (L.) Raf.

Yihao Kang [1,2], Gratien Twagirayezu [3,4], Jie Xu [1], Yunying Wen [1,2], Pengxiang Shang [2], Juan Song [1], Qian Wang [1], Xianliang Li [5], Shengqiu Liu [5], Tingsu Chen [1], Tong Cheng [6], Jinlian Zhang [1,*]

*Corresponding author,

Jinlian Zhang

Address: School of Life Sciences, Xiamen University, Xiamen, Fujian 361102, China

Email: zhangjl01@gxaas.net

Tel: +86-13006919006

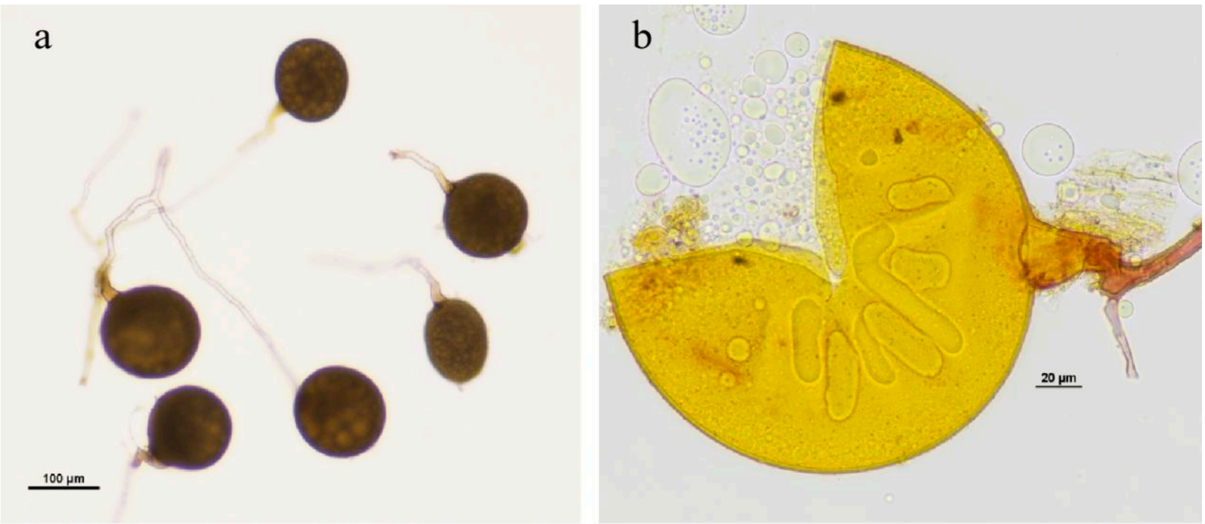

Figure S1. *F. mosseae* spores. a. Intact spores in water， b. Spore wall layers and subtending hyphain Melzer's reagent.

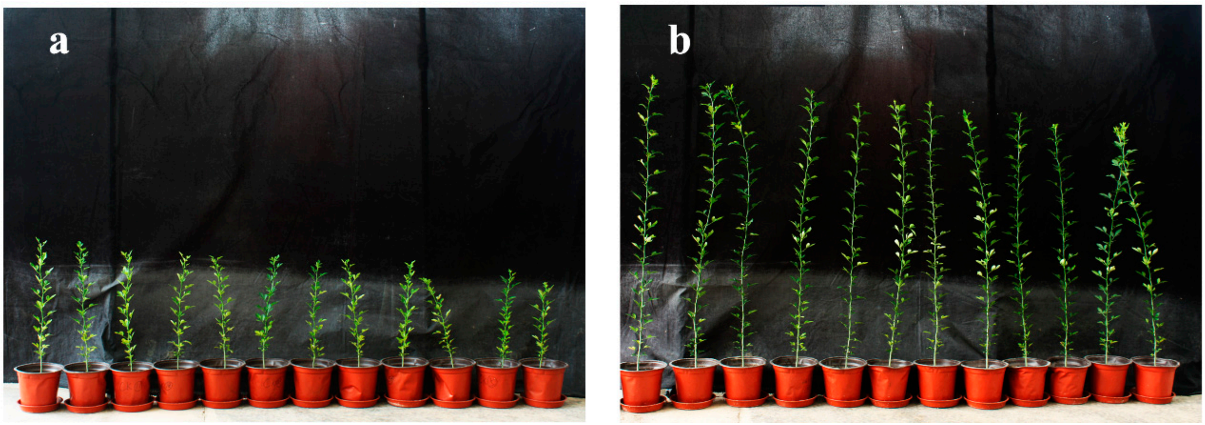

Figure S2. The growth of orange *P. trifoliata* in CK treatment (a) and AMF treatment (b). The labels of CK and AMF represent without (control) and with Arbuscular mycorrhizal fungi (*F. mosseae*).

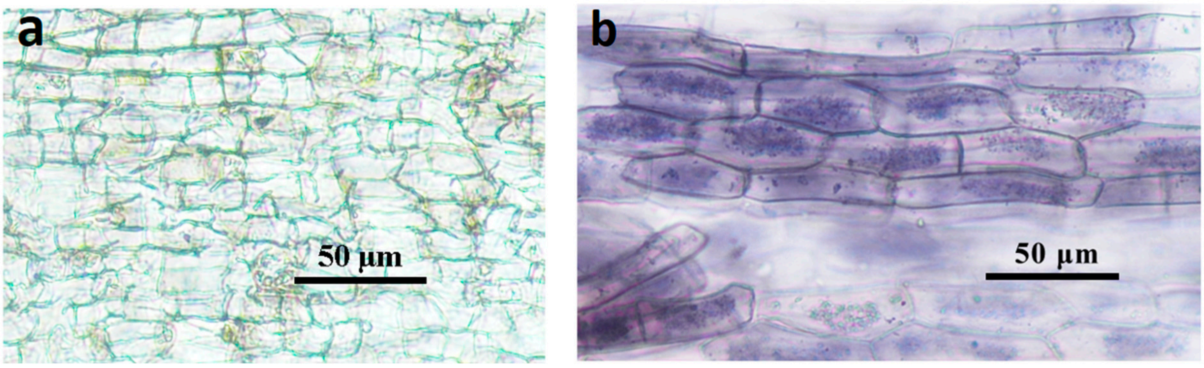

Figure S3. Visualization of *P. trifoliata* root system in the CK treatment (a) and AMF treatment (b). The labels of CK and AMF represent without (control) and with Arbuscular mycorrhizal fungi (*F. mosseae*).

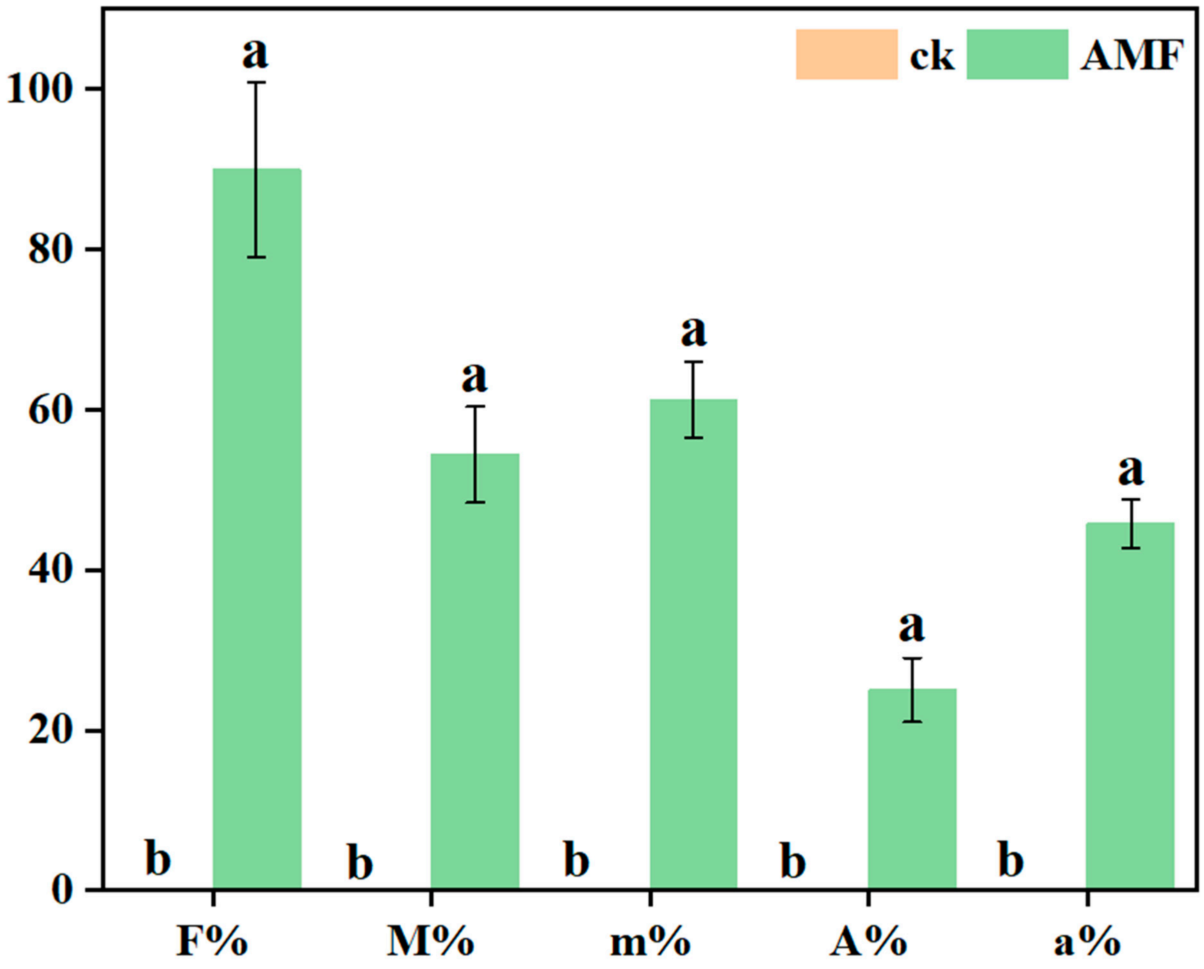

Figure S4. Mycorrhizal colonization and arbuscular abundance. F%, M%, m%, A% and a% represent Infection rate, Infection intensity, Relative infection intensity, Arbuscular abundance, and Relative arbuscular abundance. All data are presented as the mean ± standard deviation (n = 6). The significant difference (Duncan test, $p < 0.05$) between two groups is represented by the superscript letters above the error bars.
